# Supplementary material for: Signatures of transposon-mediated genome inflation, host specialization, and photoentrainment in Entomophthora muscae and allied entomophthoralean fungi
Source: bioRxiv. 2024 Mar 9:2023.09.13.557621. Originally published 2023 Sep 16. Preprint. [Version 2] doi: 10.1101/2023.09.13.557621 (PMC10515909; doi:10.1101/2023.09.13.557621)
Supplement: 4 [file NIHPP2023.09.13.557621v2-supplement-4.pdf]

bioRxiv preprint doi: https://doi.org/10.1101/2023.09.13.557621; this version posted March 9, 2024. The copyright holder for this preprint (which was not certified by peer review) is the author/funder, who has granted bioRxiv a license to display the preprint in perpetuity. It is made available under aCC-BY 4.0 International license.

Supplementary materials

Supplemental Table 1. Variance in predicted gene models using different annotation pipelines does not explain large gene count predicted in *E. muscae* genome.

Species	Gene models - original pipeline	Gene models - funannotate pipeline
Entomophaga maimaiga	14,701*	23,807
Z. radicans	14,479**	18,761
M. cicadina	N/A	7,532

*FilteredModels3, per https://mycocosm.jgi.doe.gov/Entmai1/Entmai1.info.html

**FilteredModels2, per https://mycocosm.jgi.doe.gov/Zoorad1/Zoorad1.info.html

bioRxiv preprint doi: https://doi.org/10.1101/2023.09.13.557621; this version posted March 9, 2024. The copyright holder for this preprint (which was not certified by peer review) is the author/funder, who has granted bioRxiv a license to display the preprint in perpetuity. It is made available under aCC-BY 4.0 International license.

Supplemental Table 2. Summary of fungal isolates and data used.

Supplemental Table 3. Information about strains used in phylogenetic and morphologic studies (related to Fig. 6).

Supplemental Table 4. SRA accession numbers of *E. muscae* RNAseq data (NCBI GSE111046) used for pooled expression analysis (related to Fig. 3).

Accession	Time_point	Tissue	Treatment
SRR6766803	72 hours	Whole fly	Exposed
SRR6766804	72 hours	Whole fly	Exposed
SRR6766805	72 hours	Whole fly	Exposed
SRR6766806	24 hours	Whole fly	Exposed
SRR6766807	24 hours	Whole fly	Exposed
SRR6766808	24 hours	Whole fly	Exposed
SRR6766809	48 hours	Whole fly	Exposed
SRR6766810	48 hours	Whole fly	Exposed
SRR6766811	48 hours	Whole fly	Exposed
SRR6766812	96 hours	Whole fly	Exposed
SRR6766813	96 hours	Whole fly	Exposed
SRR6766814	96 hours	Whole fly	Exposed
SRR6766815	48 hours	Whole fly	Exposed
SRR6766816	48 hours	Whole fly	Exposed
SRR6766817	48 hours	Whole fly	Exposed
SRR6766818	24 hours	Whole fly	Exposed
SRR6766819	24 hours	Whole fly	Exposed
SRR6766820	72 hours	Whole fly	Exposed
SRR6766821	24 hours	Whole fly	Exposed
SRR6766822	72 hours	Whole fly	Exposed
SRR6766823	72 hours	Whole fly	Exposed
SRR6766824	96 hours	Whole fly	Infected cadaver
SRR6766825	96 hours	Whole fly	Infected cadaver
SRR6766826	96 hours	Whole fly	Infected cadaver
SRR6766827	120 hours	Whole fly	Infected cadaver
SRR6766828	120 hours	Whole fly	Infected cadaver
SRR6766829	120 hours	Whole fly	Infected cadaver

bioRxiv preprint doi: https://doi.org/10.1101/2023.09.13.557621; this version posted March 9, 2024. The copyright holder for this preprint (which was not certified by peer review) is the author/funder, who has granted bioRxiv a license to display the preprint in perpetuity. It is made available under aCC-BY 4.0 International license.

File S1. Genome sizes and gene counts across Fungi (related to Fig. 2).

Zenodo: E. muscae genome annotation repository

Zenodo: E.muscae comparative repository

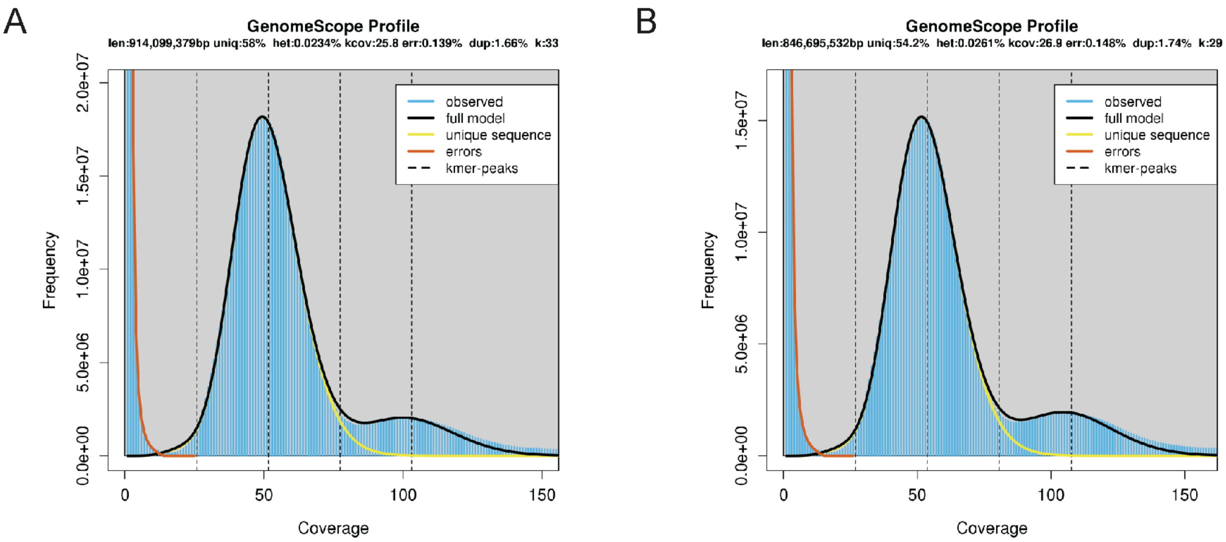

Figure. 1-S1. Kmer distributions within *E. muscae* genome assembly. A) 33mers; B) 29mers. Jellyfish was used to count kmers and plots were generated with GenomeScope. "Len" indicates estimated assembly size (in bp); "uniq": % of unique kmers observed; "kcov": estimated coverage of assembly; "err": % error kmers; "dup": %duplicated kmers; "het": indicates % of heterozygous bases; "k": kmer size.

bioRxiv preprint doi: https://doi.org/10.1101/2023.09.13.557621; this version posted March 9, 2024. The copyright holder for this preprint (which was not certified by peer review) is the author/funder, who has granted bioRxiv a license to display the preprint in perpetuity. It is made available under aCC-BY 4.0 International license.

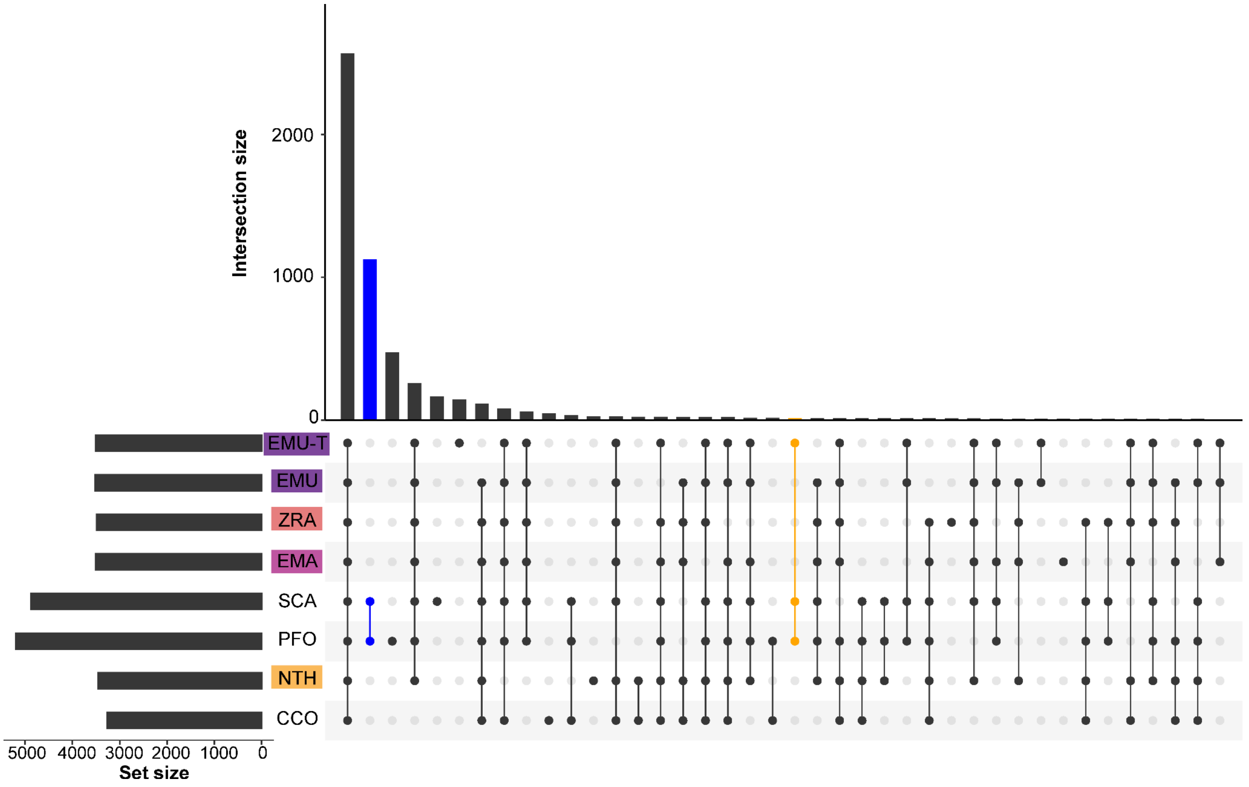

Figure 3-S1. Pfam UpSet plot analysis including *E. muscae* transcriptome. UpSet plots displaying Pfam domain intersections including additional predictions from an *E. muscae* transcriptomic dataset (EMU-T; NCBI GSE111046), showing the intersection among included genomes and transcriptomes. This analysis compares the genome predictions (i.e., EMU, EMA, ZRA, CTH and CCO) to the transcriptome predictions (i.e., EMU-T, SCA and PFO). Blue highlights the Pfam domains uniquely shared by SCA and PFO. Orange highlights the Pfam domains uniquely shared by all transcriptomic datasets in this analysis.

bioRxiv preprint doi: https://doi.org/10.1101/2023.09.13.557621; this version posted March 9, 2024. The copyright holder for this preprint (which was not certified by peer review) is the author/funder, who has granted bioRxiv a license to display the preprint in perpetuity. It is made available under aCC-BY 4.0 International license.

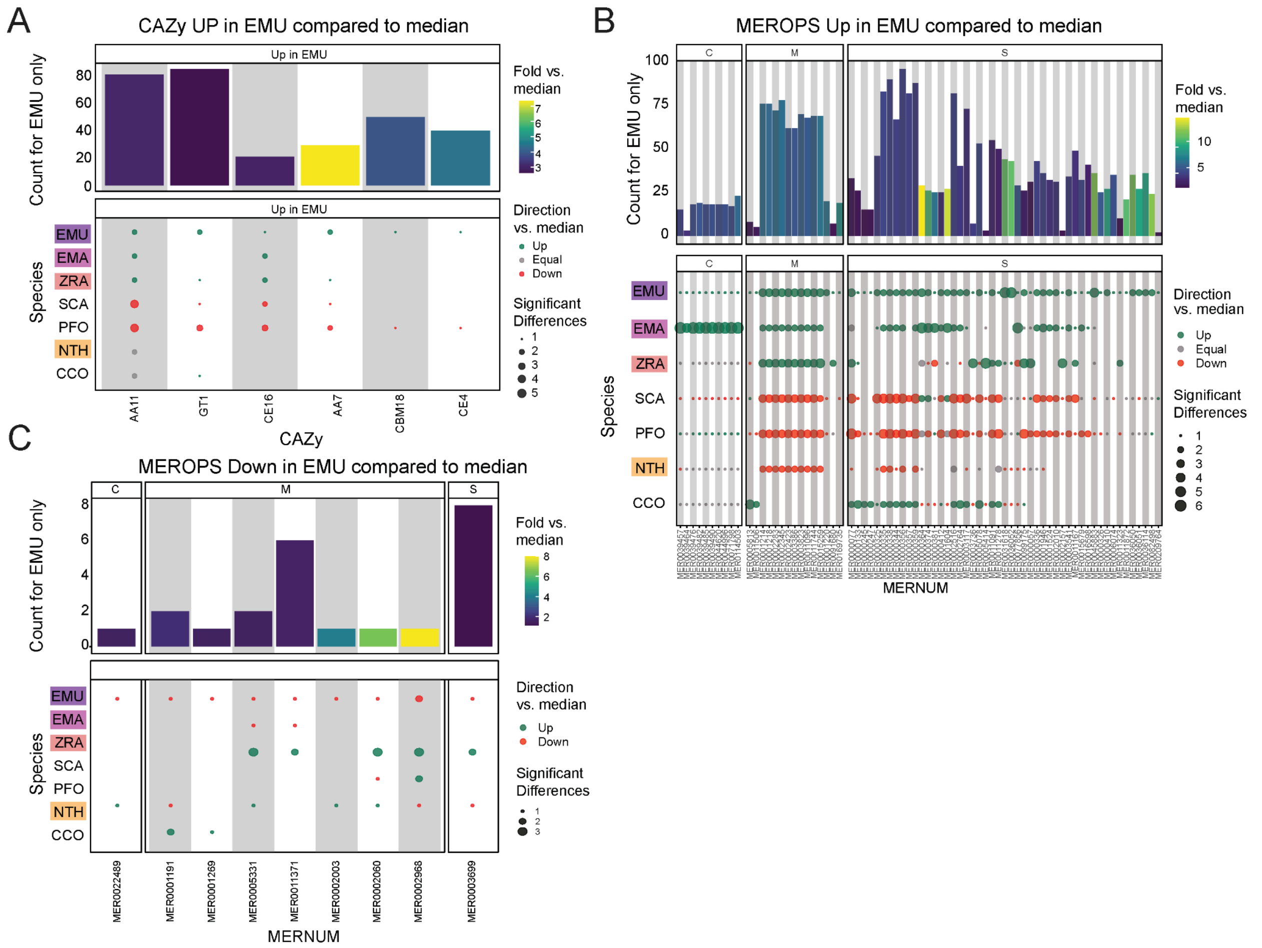

Figure 3-S2. Additional domain analysis for CAZy and MEROPS databases. A) CAZy domains significantly overrepresented in *E. muscae* (EMU) compared to other species analyzed (*E. maimaiga* (EMA), *Z. radicans* (ZRA), *S. castrans* (SCA), *P. formicae* (PFO), *N. thromboides* (CTH) and *C. coronatus* (CCO). Bars represent the counts for *E. muscae* colored by fold-versus-the-median across all genomes. Point size represents the number of significant pairwise comparisons among other genomes and are colored according to whether the value is above, below or equal to the median value across all genomes. B) Plots following a similar format as panel A, displaying MEROPS domains that were found to be overrepresented in EMU by comparison, categorized by MEROPS peptidase category (C: cysteine peptidases, M: metallopeptidases, S: serine peptidases). C) Plot following a similar format to panel B, displaying MEROPS domains that were found to be underrepresented in EMU by comparison.

bioRxiv preprint doi: https://doi.org/10.1101/2023.09.13.557621; this version posted March 9, 2024. The copyright holder for this preprint (which was not certified by peer review) is the author/funder, who has granted bioRxiv a license to display the preprint in perpetuity. It is made available under aCC-BY 4.0 International license.

PFAM	Odds-ratio	-log10(pval)	Functional annotation
PF00187	0.00	3.86	Chitin recognition protein
PF02458	0.00	3.86	Transferase family
PF00394	0.00	3.41	Multicopper oxidase
PF01522	0.00	3.39	Polysaccharide deacetylase
PF07732	0.00	3.19	Multicopper oxidase
PF03732	0.05	5.16	Retrotransposon gag protein
PF16187	0.11	4.33	Middle or third domain of peptidase_M16
PF00001	0.11	6.10	7 transmembrane receptor (rhodopsin family)
PF03982	0.14	3.15	Diacylglycerol acyltransferase
PF01764	0.20	11.41	Lipase (class 3)
PF00675	0.29	3.60	Insulinase (Peptidase family M16)
PF05193	0.31	3.91	Peptidase M16 inactive domain
PF00089	0.32	6.75	Trypsin
PF00046	0.38	3.39	Homeodomain
None	0.46	231.72	N/A
PF00067	0.49	3.27	Cytochrome P450
PF00172	0.55	3.09	Fungal Zn(2)-Cys(6) binuclear cluster domain
PF00069	1.52	5.51	Protein kinase domain
PF07714	1.54	5.42	Protein tyrosine and serine/threonine kinase
PF00076	1.56	3.49	RNA recognition motif
PF00004	1.76	4.12	ATPase family (AAA)
PF00400	1.79	9.60	WD domain_ G-beta repeat
PF00270	1.85	5.42	DEAD/DEAH box helicase
PF04851	1.88	3.91	Type III restriction enzyme_ res subunit
PF00271	1.92	7.32	Helicase conserved C-terminal domain
PF00169	2.20	4.61	PH domain
PF00176	2.22	3.00	SNF2-related domain
PF00226	2.24	3.54	DnaJ domain
PF00621	2.35	3.63	RhoGEF domain

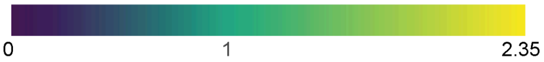

Figure 3-S3. *E. muscae* core OG Pfam enrichment. Enrichment among Pfam annotations for *E. muscae* genes belonging to core OG set. Odds-ratios are colored according to the scale bar below.

bioRxiv preprint doi: https://doi.org/10.1101/2023.09.13.557621; this version posted March 9, 2024. The copyright holder for this preprint (which was not certified by peer review) is the author/funder, who has granted bioRxiv a license to display the preprint in perpetuity. It is made available under aCC-BY 4.0 International license.

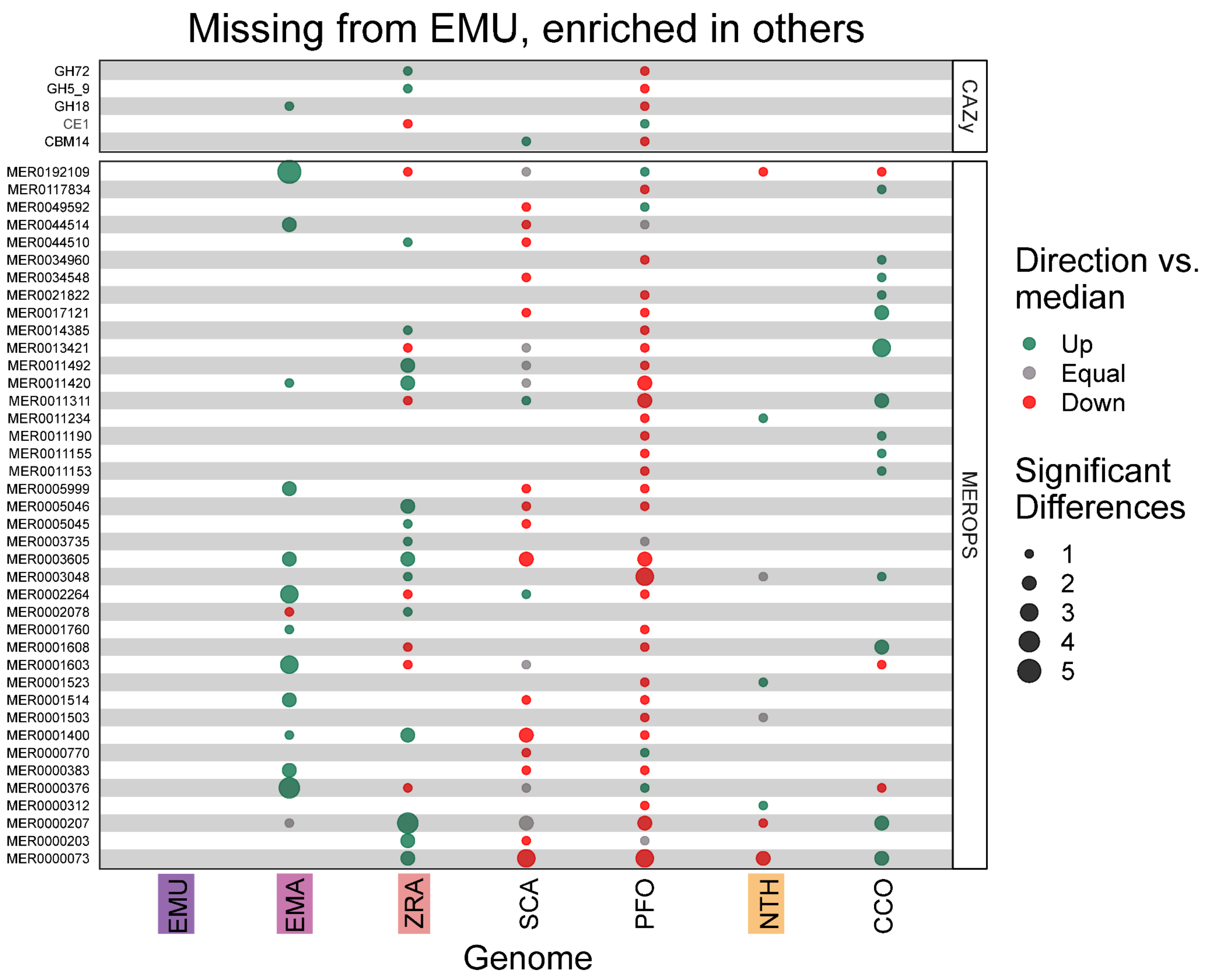

Figure 5-S1. CAZy and MEROPs domains missing in *E. muscae* and enriched in other fungi. CAZy and MEROPS domains missing from *E. muscae* (EMU), but significantly underrepresented (red) or overrepresented (green) in other species analyzed (*E. maimaiga* (EMA), *Z. radicans* (ZRA), *S. castrans* (SCA), *P. formicae* (PFO), *N. thromboides* (CTH) and *C. coronatus* (CCO).
